# Supplementary material for: Complementary Feeding: Pitfalls for Health Outcomes
Source: Int J Environ Res Public Health. 2020 Oct 29;17(21):7931. doi: 10.3390/ijerph17217931 (PMC7662522; doi:10.3390/ijerph17217931)
Supplement: Supplementary file 1 [file ijerph-17-07931-s001.pdf]

TableS1: Recommended daily intake for macro and micronutrients during the first year of life

	**Italian Recommended Dietary Allowances (RDA)**		**UK British Nutrition Foundation**		**EFSA (European Food Safety Authority)**		**US NIH (National Institutes of Health)**	
Energy	6 mo<br>7 mo<br>8 mo<br>9 mo<br>10 mo<br>11 mo<br>12 mo	620 kcal/d (M)<br>570 kcal/d (F)<br>640 kcal/d (M)<br>580 kcal/d (F)<br>660 kcal/d (M)<br>600 kcal/d (F)<br>690 kcal/d (M)<br>630 kcal/d (F)<br>730 kcal/d (M)<br>640 kcal/d (F)<br>740 kcal/day (M)<br>660 kcal/day (F)<br>790 kcal/day (M)<br>690 kcal/day (F)	1-2 mo<br>3-4 mo<br>5-6 mo<br>7-12 mo	574 kcal/d (M)<br>502 kcal/d (F)<br>598 kcal/d (M)<br>550 kcal/d (F)<br>622 kcal/d (M)<br>574 kcal/d (M)<br>718 kcal/d (M)<br>646 kcal/d (M)	6 mo<br>7 mo<br>8 mo<br>9 mo<br>10 mo<br>11 mo<br>12 mo	597 kcal/d (M) (AR)<br>549 kcal/d (F) (AR)<br>636 kcal/d (M) (AR)<br>573 kcal/d (F) (AR)<br>661 kcal/d (M) (AR)<br>599 kcal/d (F) (AR)<br>688 kcal/d (M) (AR)<br>625 kcal/d (F) (AR)<br>725 kcal/d (M) (AR)<br>656 kcal/d (F) (AR)<br>742 kcal/d (M) (AR)<br>673 kcal/d (F) (AR)<br>777 kcal/d (M) (AR)<br>712 kcal/d (F) (AR)	-	-
Total water	6-12 mo	0.8 L/d (AI)	-	-	7-11 mo	0.8 L/d (AI)	0-6 mo<br>6-12 mo	0.7 L/d (AI)<br>0.8 L/d (AI)
Carbohydrates	All ages	45-60 % E	-	-	7-11 mo	NA	0-6 mo<br>6-12 mo	60 g/d (AI)<br>95 g/d (AI)
Soluble sugars	All ages	<15% E	-	-	-	-		-
Dietary fiber	-	-	-	-	7-11 mo	ND	0-6<br>6-12 mo	ND
Total lipids	0-12 mo	40% E (AI)	-	-	7-11 mo	40 % E (AI)	0-6 mo<br>6-12 mo	31 g/d (AI)<br>30 g/d (AI)
Alpha-linolenic acid (ALA)	-	-	-	-	7-11 mo	0.5 % E (AI)	0-6 mo<br>6-12 mo	0.5 g/d (AI)<br>0.5 g/d (AI)
Eicosapentaenoic acid, Docosahexaenoic acid (EPA, DHA)	0-12 mo	EPA-DHA 250 mg + DHA 100 mg	-	-	7-11 mo	100 mg/d DHA (AI)	-	-
Linoleic acid (LA)	-	-	-	-	7-11 mo	4 % E	0-6 mo<br>6-12 mo	4.4 g/d (AI)<br>4.6 g/d (AI)
Saturated fatty acids (SFA)	0-12 mo	<10% E	-	-	7-11 mo	ALAP (AI)	-	-
Trans-fatty acids (TFA)	0-12 mo	ALAP	-	-	7-11 mo	ALAP (AI)	-	-
Proteins	6-12 mo	1.11 g/kg bw per d (AR)<br>1.32 g/kg bw per d (PRI)	0-3 mo<br>4-6 mo<br>7-9 mo<br>10-12 mo	12.5 g/d<br>12.7 g/d<br>13.7 g/d<br>14.9 g/d	7-11 mo	1.12 g/kg bw per d (AR)<br>1.31 g/kg bw per d (PRI)	0-6 mo<br>6-12 mo	9.1 g/d (AI)<br>11 g/d (RDA)
**Minerals**								

Calcium	6-12 mo	260 mg/d (AI)	0-3 mo<br>4-6 mo<br>7-9 mo<br>10-12 mo	525 mg/d<br>525 mg/d<br>525 mg/d<br>525 mg/d	7-11 mo	280 mg/d (AI)	0-6 mo<br>7-12 mo	200 mg/d (AI)<br>260 mg/d (AI)
Iron	6 - 12 mo	11 mg/d (PRI)	0-3 mo<br>4-6 mo<br>7-9 mo<br>10-12 mo	1.7 mg/d<br>4.3 mg/d<br>7.8 mg/d<br>7.8 mg/d	7-11 mo	8 mg/d (AR)<br>11 mg/d (PRI)	0-6 mo<br>7-12 mo	0.27 mg/d (AI)<br>11 mg/d (RDA)
Sodium	6 - 12 mo	0.4 g/d (AI)	0-3 mo<br>4-6 mo<br>7-9 mo<br>10-12 mo	210 mg/d<br>280 mg/d<br>320 mg/d<br>350 mg/d	7-11 mo	0.2 g/d (AI)	0-6 mo<br>7-12 mo	110 mg/d (AI)<br>370 mg/d (AI)
Zinc	6 - 12 mo	3 mg/d (AI)	0-3 mo<br>4-6 mo<br>7-9 mo<br>10-12 mo	4 mg/d<br>4 mg/d<br>5 mg/d<br>5 mg/d	7-11 mo	2.4 mg/d (AR)<br>2.9 mg/d (PRI)	0-6 mo<br>7-12 mo	2 mg/d (AI)<br>3 mg/d (RDA)
**Vitamins**								
Cobalamin (vitamin B12)	6 - 12 mo	0.7 µg/d (AI)	0-3 mo<br>4-6 mo<br>7-9 mo<br>10-12 mo	0.3 µg/d<br>0.3 µg/d<br>0.4 µg/d<br>0.4 µg/d	7-11 mo	1.5 µg/d (AI)	0-6 mo<br>6-12 mo	0.4 µg/d (AI)<br>0.5 µg/d (AI)
Folate	6 - 12 mo	110 µg/d (AI)	0-3 mo<br>4-6 mo<br>7-9 mo<br>10-12 mo	50 µg/d<br>50 µg/d<br>50 µg/d<br>50 µg/d	7-11 mo	80 µg DFE/d (AI)	0-6 mo<br>6-12 mo	65 µg/d (AI)<br>80 µg/d (AI)
Vitamin A	6 - 12 mo	450 µg/d (AI)	0-3 mo<br>4-6 mo<br>7-9 mo<br>10-12 mo	350 µg/d<br>350 µg/d<br>350 µg/d<br>350 µg/d	7-11 mo	190 µg/d RE/d (AR)<br>250 µg/d RE/d (PRI)	0-6 mo<br>6-12 mo	400 µg/d (AI)<br>500 µg/d (AI)
Vitamin D	6-12 mo	10 µg/d (AI)	0-3 mo<br>4-6 mo<br>7-9 mo<br>10-12 mo	8.5 – 10 µg/d<br>8.5 – 10 µg/d<br>8.5 – 10 µg/d<br>8.5 – 10 µg/d	7-11 mo	10 µg/d (AI)	0-6 mo<br>6-12 mo	10 µg/d (AI)<br>10 µg/d (AI)

Legend:
ND not determined
NA not available
AI adequate intakes
AR average requirement
RDA recommended dietary allowances
ALAP as low as possible
DFE dietary folate equivalents
NE niacin equivalent
RE retinol equivalent
- not specified
